# Cumulative cerebral desaturation burden during cardiopulmonary bypass predicts postoperative S100B elevation in pediatric cardiac surgery: a prospective cohort study

**DOI:** 10.3389/fped.2026.1922477

**Published:** 2026-09-14

**Authors:** Xiaoxue Wang, Zhengzheng Gao, Lei Hua, Zhangke Guo, Fang Wang, Lijing Li, Jianmin Zhang

**Affiliations:** 1 Department of Anesthesiology, Beijing Children's Hospital, Capital Medical University, National Center for Children's Health, Beijing, China; 2 Department of Cardiac Surgery, Beijing Children's Hospital, Capital Medical University, National Center for Children's Health, Beijing, China

**Keywords:** brain injury, cardiopulmonary bypass, near-infrared spectroscopy, regional oxygen saturation, S100B

## Abstract

**Background:**

Pediatric cardiac surgery with cardiopulmonary bypass (CPB) carries a high risk of perioperative brain injury, while the optimal threshold of cerebral regional oxygen saturation (C-rSO₂) for early risk identification remains unclear. This study aimed to investigate the association between cumulative cerebral desaturation burden during CPB and perioperative serum S100B levels in children undergoing congenital heart surgery, and to evaluate the consistency of this association across four relative desaturation thresholds via sensitivity analysis.

**Methods:**

In this prospective cohort study, intraoperative C-rSO₂ was continuously monitored, and the area under the curve (AUC) for C-rSO₂ below baseline (5%, 10%, 15%, 20% below individual baseline) during CPB was calculated. Serum S100B was measured at anesthesia induction (T0, baseline), end of surgery (T1), and first postoperative day (T2). Multiple linear regression models adjusted for confounders were compared, and receiver operating characteristic (ROC) curve analysis was performed to explore the optimal cutoff value.

**Results:**

A total of 118 children were finally included. After adjusting for confounders, the association between cumulative desaturation burden and S100B elevation was consistent across all tested thresholds, with the ≥5% threshold being the most sensitive predictor at both T1 (B = 0.22, 95%CI 0.19–0.25, *P* < 0.001) and T2 (B = 0.15, 95%CI 0.12–0.18, *P* < 0.001), with the best model performance (adjusted R_2_: 0.80 at T1, 0.79 at T2). ROC analysis showed an AUROC of 0.87 (95%CI: 0.78–0.97) for predicting elevated peak S100B, with an optimal cutoff of 217.1%·min (sensitivity 86.2%, specificity 84.3%). A significant dose-response relationship was observed, and both markers were elevated in children with postoperative seizures.

**Conclusion:**

Cumulative C-rSO₂ desaturation burden ≥5% during CPB is a robust independent predictor of early postoperative S100B elevation.

## Introduction

Despite improved survival after cardiopulmonary bypass (CPB) for congenital heart disease (CHD) repair, children remain at substantial risk of neurological impairment. Acute severe neurological injury occurs in 4%–8% of these children, and long-term neurodevelopmental abnormalities affect 40%–50% of school-aged children (1–3). The developing pediatric brain is particularly vulnerable: neonates, infants, and young children exhibit a 30% higher cerebral metabolic rate than adults, increasing susceptibility to ischemia and hypoxia (4). Seizures represent the most common neurological complication after pediatric CHD open surgery, with an incidence of 1.4%–7.2% (5–8). Most early postoperative neurological injuries are subclinical. Conventional clinical assessments fail to promptly identify subtle brain damage, resulting in missed optimal intervention windows.

Postoperative neurological injury is currently diagnosed relying on clinical neurological evaluation, whose accuracy depends on exhaustive functional assessment of memory, language, reading, and cognition. However, such comprehensive assessments are rarely performed and are technically challenging in groups of young children. Moreover, these evaluations are conducted retrospectively, only detecting injury after irreversible damage has formed. Accordingly, reliable early biomarkers for pre-emptive prediction of neurological injury are urgently needed. S100B, a calcium-binding protein predominantly expressed in cerebral astrocytes, is a damage-associated molecular pattern released upon primary brain injury and mediates secondary brain damage (9). As a biomarker of brain injury, S100B levels have been correlated with the extent of injury, survival rates, and neurological outcomes (10).

The biological functions of S100B are concentration-dependent. Physiological S100B levels exert neurotrophic effects to facilitate neuronal development and repair (11–13) whereas pathologically elevated S100B induces neurotoxicity and neuronal apoptosis (14). Astrocyte activation and blood-brain barrier disruption are major causes of marked S100B elevation in serum and cerebrospinal fluid after brain injury (15).

Despite its clinical value for confirming glial injury, S100B cannot provide real-time intraoperative risk alerts. By contrast, near-infrared spectroscopy (NIRS) enables continuous, non-invasive intraoperative monitoring of cerebral regional oxygen saturation (C-rSO₂), allowing real-time detection of cerebral hypoxia prior to irreversible biochemical brain injury. A validated predictive relationship between intraoperative NIRS-detected cerebral desaturation and postoperative S100B elevation would establish NIRS as a promising early warning tool to guide timely neuroprotective interventions.

The study objectives were to: (1) explore the association between CPB-related cumulative C-rSO₂ desaturation burden and postoperative serum S100B levels in children with CHD; (2) verify the stability and magnitude of this association and determine the optimal relative desaturation threshold through sensitivity analysis; (3) investigate the correlations of C-rSO₂ parameters and S100B levels with short-term clinical outcomes.

## Methods

### Study design

This prospective observational cohort study was approved by the Institutional Review Board of Beijing Children's Hospital [Approval ID: (2024)-Y-093-D; Approval date: March 29, 2024] and prospectively registered with the Chinese Clinical Trial Registry (Registration number: ChiCTR2400083225). The study was conducted in strict compliance with the ethical principles outlined in the Declaration of Helsinki and relevant clinical practice guidelines. Prior to study initiation, written informed consent was obtained through structured discussions with parents or legal guardians of all eligible participants.

### Children and eligibility criteria

Between April and November 2024, children scheduled to receive elective cardiac surgery underwent eligibility screening for enrollment. Inclusion criteria: (1) Age ≤6 years at enrollment. (2) Scheduled to undergo corrective congenital heart disease surgery under CPB. (3) Written informed consent obtained from parents or legal guardians. Exclusion criteria: (1) Premature infants with postmenstrual age ≤38 weeks. (2) Documented neurodevelopmental disorders (autism spectrum disorder, global developmental delay, or diagnosed cognitive impairment). (3) History of any following conditions: prior heart transplantation, active systemic infection or sepsis, preoperative extracorporeal life support.

### Study procedures

All children underwent standard anesthetic management for their surgical procedure, administered by a trained pediatric anesthesiologist. From admission to the operating room until transfer to the pediatric cardiac intensive care unit (CICU) postoperatively, continuous C-rSO₂ monitoring was performed using the SenSmart™ Model X-100 Universal Oximetry System (Nonin Medical, Inc., Plymouth, MN, USA), with measurements recorded at 4-second intervals. During mechanical ventilation in the pre-CPB and post-CPB periods, the fraction of inspired oxygen (FiO₂) was maintained at 1.0. The CPB oxygenation strategy is described in detail in the Standard CPB Techniques section below. Prior to sensor electrode placement, the forehead skin condition of each patient was evaluated by the study team to ensure optimal adhesion and signal quality. A trained member of the study team placed the single-channel NIRS sensor on the midline of the patient's forehead, over the frontal region corresponding to the anterior cerebral artery territory, and connected it to the monitoring device to initiate continuous C-rSO₂ data acquisition. This single midline frontal configuration is a standard, validated approach in pediatric cardiac surgery, providing stable signal acquisition in infants and reliably reflecting global cerebral oxygenation trends (16, 17). To prevent electrode displacement, the sensor was secured with an elastic bandage if necessary.

During anesthetic induction, children with established intravenous (IV) access received an intravenous bolus of propofol, whereas those without pre-existing vascular access underwent mask-assisted inhalational induction with sevoflurane. All children underwent standardized endotracheal intubation for airway management. Anesthesia was maintained with continuous inhalation of sevoflurane, supplemented by intermittent boluses of opioids and neuromuscular blocking agents. Pharmacological regimens, including the administration of vasoactive agents, were individualized by the attending anesthesiologists based on real-time intraoperative hemodynamic and clinical needs.

### Standard CPB techniques

All children were managed with standardized CPB protocols. Hemoglobin Targets: Age-stratified hemoglobin targets were adopted: neonates and infants (<10 kg) 90–100 g/L, and children >10 kg 80–90 g/L (with a permissible lower limit of 70 g/L during deep hypothermia). Transfusion Triggers: Transfusion triggers were dynamically titrated based on continuous monitoring of venous oxygen saturation (SvO₂; target >65%) and lactate levels (target <2.5 mmol/L). Perfusion Parameters: CPB pump flow rates were adjusted according to core body temperature: 150–200 mL/kg/min during normothermia (36–37 °C); 100–150 mL/kg/min during moderate hypothermia (28–32 °C); and 50–80 mL/kg/min during deep hypothermia (<28 °C). Mean arterial pressure (MAP) was maintained within an age-appropriate range of 30–50 mmHg. Oxygenation Strategy: Normoxic oxygenation targets [arterial oxygen saturation (SaO₂); 95%–98%] were adopted instead of hyperoxic strategies, with permissive desaturation (SaO₂ 75%–85%) permitted during deep hypothermic circulatory arrest. Ultrafiltration: Combined conventional ultrafiltration (CUF; 80–120 mL/min for 8 min) and modified ultrafiltration (MUF; 30–50 mL/kg initiated post-CPB) were conducted via dedicated circuits to avoid interference with cardioplegia catheters.

### Measurement of C-rSO_2_

C-rSO₂ monitoring data were electronically recorded, with no real-time clinical disclosure to the attending anesthesiologist or perfusionist to avoid interference with clinical decision-making. A standardized quality control protocol was applied to all raw C-rSO₂ recordings prior to formal analysis. First, measurements were filtered according to the built-in signal quality index (SQI) of the SenSmart™ X-100 system; only readings classified as “good” or “fair” signal quality were retained, while “poor” quality signals were excluded. Second, predefined artifact rejection rules were applied to remove physiologically implausible values, including: (1) abrupt changes greater than 15% within a single 4-second sampling interval; (2) values outside the physiological range of 15% to 95%; and (3) time periods with documented sensor displacement per intraoperative study logs. For missing data intervals, gaps shorter than 30 s were filled using linear interpolation, whereas gaps longer than 30 s were excluded from subsequent AUC calculations. No additional data smoothing was applied to the raw 4-second sampling data, to preserve the temporal precision of desaturation burden quantification. All NIRS data processing and AUC calculations were performed by dedicated research personnel who were fully blinded to postoperative S100B concentrations and all clinical outcome data. Data preprocessing was completed before the biomarker database was unlocked for statistical analysis.

The study protocol defined three distinct phases for C-rSO₂ analysis: (1) pre-CPB phase; (2) intra-CPB phase (from CPB initiation to weaning); and (3) post-CPB phase (from CPB termination to surgical closure). Baseline cerebral regional oxygen saturation was defined as the mean of all valid C-rSO₂ recordings obtained throughout the entire pre-CPB period, from the completion of endotracheal intubation to the initiation of CPB. Mean C-rSO₂ values for each phase were computed by temporal averaging of all valid measurements within the respective time intervals. Four cerebral desaturation thresholds were defined relative to the individual baseline: ≥5%, ≥10%, ≥15%, and ≥20% decrease from baseline. All four desaturation thresholds represent relative percentage decreases from each child's individual baseline C-rSO₂, rather than absolute percentage-point decreases in saturation value. For each time point t, the relative desaturation magnitude was calculated as: Relative decrease (%) = {[Baseline C-rSO₂−C-rSO₂(t)]/Baseline C-rSO₂}  ×  100%. For a child with a baseline C-rSO₂ of 80% and an intraoperative value of 72% at time t: Relative decrease = [(80−72)/80] × 100% = 10%. This observation would count toward both the ≥5% and ≥10% desaturation thresholds. Cumulative desaturation burden was quantified as the area under the curve (AUC) of relative desaturation below each threshold during CPB, calculated using the linear trapezoidal method, with units of %·min. This metric integrates both the depth and duration of desaturation to quantify cumulative cerebral hypoxic burden. Cerebral oximetry monitoring was implemented as an additional research-specific measurement and was not part of routine standard care for pediatric cardiac surgery at our institution during the study period.

### Serum S100B

S100B levels were measured at three perioperative time points: after anesthesia induction (baseline, T0), immediately after surgery (T1), and on the first postoperative day (POD, T2). At each time point, 2 mL of blood was collected from the children. The baseline sample was drawn after anesthesia induction and prior to CPB initiation. The end-of-surgery sample was obtained within 5 min after the procedure. The CICU sample was collected on the first postoperative day. After collection, blood samples were allowed to clot at room temperature for 30 min. Samples were then centrifuged at 3000 rpm (approximately 1,500 g) for 10 min at 4 °C to separate serum. The upper serum layer was immediately aspirated and aliquoted into 100 μL cryovials, labeled with anonymized study ID numbers and corresponding sampling time points, and transferred immediately to a −80 °C ultra-low temperature freezer for storage until analysis. All specimens were strictly protected from repeated freeze-thaw cycles throughout storage, and each sample underwent exactly one freeze-thaw cycle immediately before detection. Serum S100B concentrations were quantified using a commercially available enzyme-linked immunosorbent assay (ELISA) kit (Human S100B ELISA Kit; Abcam, Cambridge, UK). The assay employs a sandwich immunoassay principle with two monoclonal antibodies targeting distinct epitopes on the human S100B protein for specific quantitative detection. Analytical measurement range: 15.6–1,000 pg/mL, lower limit of detection: 4.7 pg/mL, intra-assay coefficient of variation: <6.2%, inter-assay coefficient of variation: <8.5%. All serum samples were analyzed in a single assay batch to minimize inter-assay variability. Laboratory personnel performing the S100B assays were fully blinded to all NIRS monitoring data, patient clinical characteristics, and perioperative outcomes; samples were identified solely by anonymized study ID numbers throughout the testing procedure.

### Sample size calculation

Sample size was estimated by the partial *F* test for multiple linear regression, with postoperative serum S100B as the primary outcome. Based on our single-center pilot study using identical inclusion criteria and detection methods, cerebral desaturation AUC explained approximately 8% of the variance in postoperative S100B. To avoid overestimating the effect size, a conservative partial R^2^ of 0.06 was adopted. The calculation was set with a two-sided *α* of 0.05, a statistical power of 80%, and 8 covariates in the full model. A minimum of 110 children was required. With a 10% anticipated rate of missing data or protocol deviation, the planned enrollment was 122 children.

### Statistical analysis

The primary outcome was serum S100B concentration at T1, selected *a priori* as the most sensitive time point to capture acute intraoperative glial injury given the short serum half-life of S100B and its close temporal alignment with CPB termination. Serum S100B at T2 was defined as a secondary endpoint to evaluate the persistence of elevation. Exploratory outcomes included time to tracheal extubation, CICU length of stay, postoperative hospital length of stay, total hospital costs, and postoperative clinical seizure.

Separate multivariable linear regression models were constructed with S100B levels at T1 and T2 as dependent variables, respectively. Potential confounders included age, sex, body mass index (BMI), Risk Adjustment for Congenital Heart Surgery-2 (RACHS-2) category, preoperative cyanosis (peripheral oxygen saturation<90%), baseline hematocrit, preoperative left ventricular ejection fraction (LVEF), CPB duration, and mean arterial pressure during CPB. First, univariable linear regression analyses were performed to screen potential perioperative confounders. Variables with *P* < 0.05 in univariable analyses, as well as clinically important variables, were included in the multivariable models. Each C-rSO₂ desaturation AUC parameter was entered into the multivariable model separately to avoid multicollinearity. Multicollinearity was assessed using variance inflation factor (VIF), with VIF < 5 indicating no significant multicollinearity.

Threshold comparison was framed as a sensitivity analysis to assess whether the desaturation-S100B association was robust to the choice of relative cutoff, rather than an attempt to identify a single optimal threshold. To quantify the incremental explanatory value of cumulative desaturation burden beyond established predictors, we constructed three nested sequential linear models for T1 and T2 S100B separately: (1) Model 1: baseline S100B only; (2) Model 2: baseline S100B plus CPB duration; (3) Model 3: baseline S100B plus CPB duration plus AUC of C-rSO₂ decrease ≥5% during CPB. Changes in adjusted R^2^ across models were calculated to estimate the additional variance in postoperative S100B explained by cumulative desaturation burden. A sensitivity analysis was performed using a more restrictive stable baseline definition, the mean C-rSO₂ value of a 5-minute steady window 10–15 min after endotracheal intubation, to verify whether the primary association remained robust.

Model performance was compared by adjusted R^2^, Akaike Information Criterion (AIC), and Bayesian Information Criterion (BIC), with higher adjusted R^2^ and lower AIC/BIC indicating better model fit. The assumptions of linear regression were evaluated through diagnostic procedures, including Shapiro–Wilk test for residual normality, residual-versus-fitted plots for visual assessment of homoscedasticity. To assess the robustness of findings to the choice of outcome scale, sensitivity analyses were performed using natural log-transformed S100B concentrations as the dependent variable with the same set of covariates. To explore the optimal cutoff value of cumulative cerebral desaturation burden for early identification of elevated risk of S100B elevation, receiver operating characteristic (ROC) curve analysis was performed as an exploratory analysis. For exploratory ROC analysis, elevated postoperative S100B was defined as a T1 concentration above the 75th percentile of the entire cohort. The area under the ROC curve (AUROC) was calculated to evaluate the predictive performance of cumulative desaturation AUC (C-rSO₂ decrease ≥5% during CPB). The optimal cutoff value was determined by the maximum Youden's index, and the corresponding sensitivity, specificity were reported. We performed 1,000 bootstrap resamples with replacement for internal validation to quantify optimism bias. In each iteration, the optimal cutoff was re-derived independently via the Youden index, and optimism-corrected estimates of AUROC, sensitivity, and specificity were computed. The 95% percentile confidence intervals for all metrics and the cutoff were generated from the bootstrap empirical distribution. Spearman's rank correlation was used to assess the relationships between postoperative serum S100B levels and clinical outcomes, including the duration of postoperative mechanical ventilation, total hospital length of stay, and total hospitalization costs. Due to the anticipated low event rate, clinical seizure was treated as an exploratory endpoint, and no multivariable adjustment was performed.

Demographic and baseline characteristics were summarized using descriptive statistics. Continuous variables (e.g., age) were presented as mean ± standard deviation (SD) or median with interquartile range (IQR), while categorical variables (e.g., sex) were expressed as frequencies and percentages. Categorical variables were analyzed using *χ*^2^ tests or Fisher's exact tests, while continuous variables were assessed via two-tailed independent t-tests or Wilcoxon rank-sum tests, depending on data distribution and variance assumptions. A Type I error threshold of *α* = 0.05 was established for statistical significance. All statistical analyses were performed using SPSS software version 22 (SPSS, Inc., Chicago, IL, USA), with graphical representations created using GraphPad Prism version 10 (GraphPad Software Company, San Diego, CA, USA).

## Results

### Demographic and clinical variables

A total of 136 consecutive children were screened for enrollment between April and November 2024, of whom 125 met the prespecified evaluability criteria. Seven children were further excluded due to incomplete monitoring data: 3 experienced NIRS sensor displacement during the pre-CPB phase resulting in >20% missing data, 2 had extensive signal artifacts caused by electrocautery interference affecting >30% of the total recording, 1 had device malfunction with no valid data captured after CPB initiation, and 1 withdrew consent intraoperatively for reasons unrelated to the study. Ultimately, 118 children were included in the final analysis (Figure 1). Baseline characteristics were compared between the 7 excluded children and the 118 included children. No statistically significant differences were observed in age, weight, RACHS-2 category, CPB duration, or baseline S100B levels (all *P* > 0.10), indicating that data missingness was most likely random rather than systematic.

**Figure 1:**
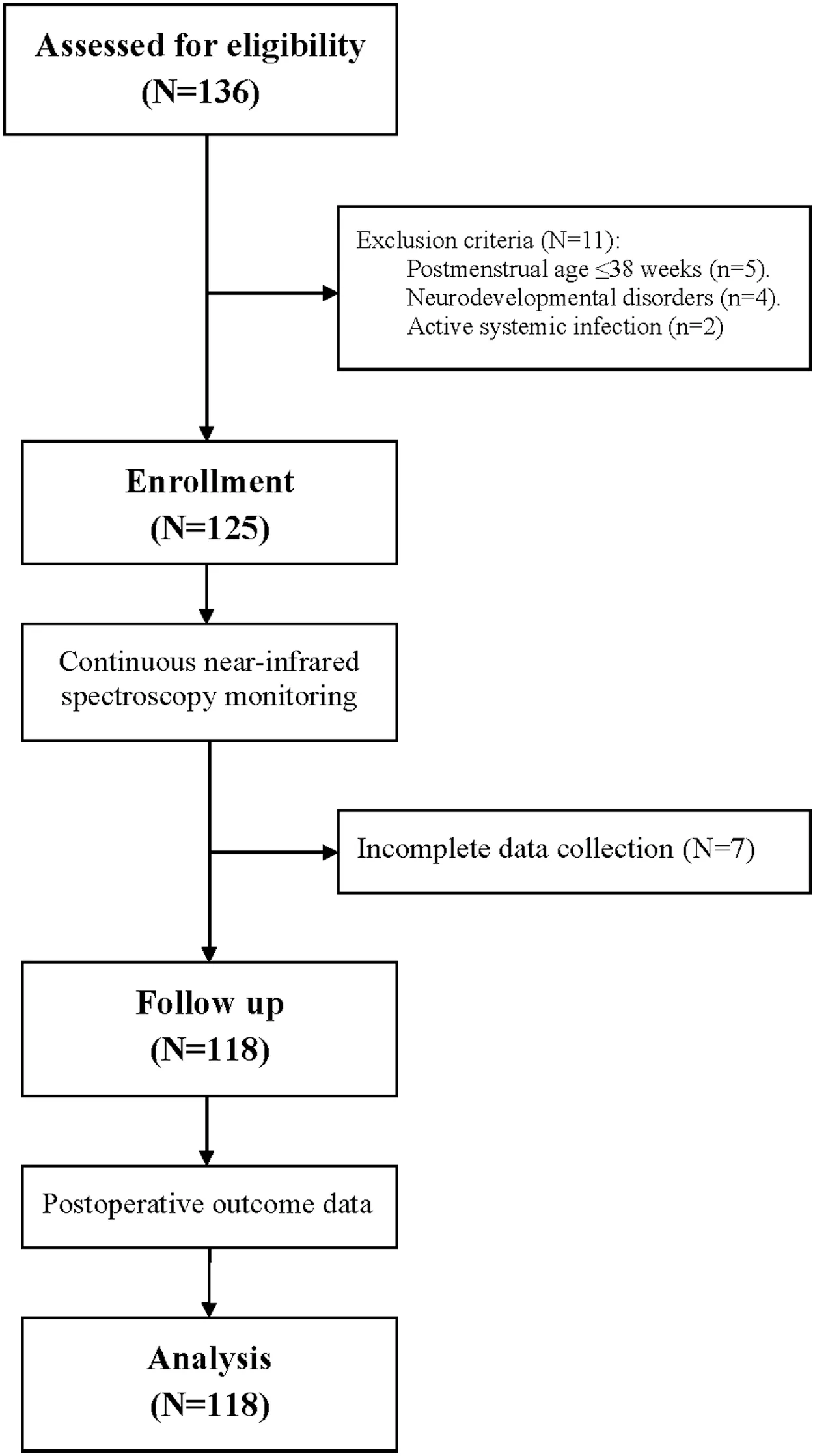
Flow diagram of the children in the study.

The final cohort consisted predominantly of infants and young children, with a median age of 19.0 months [interquartile range (IQR) 7.0–42.5 months] and a nearly equal sex distribution (50.8% male). Most children presented with acyanotic congenital heart lesions, with cyanotic disease accounting for 10.2% of the cohort. Surgical complexity was generally low, with a median Risk Adjustment for Congenital Heart Surgery-2 (RACHS-2) category of 1. Intraoperatively, the median duration of CPB was 84.5 min (IQR 67.8–101.3 min), and the median aortic cross-clamp time was 58.0 min. All included children underwent conventional full-flow CPB under standardized perfusion protocols; deep hypothermic circulatory arrest and selective cerebral perfusion were not used in this cohort. Postoperatively, the median duration of mechanical ventilation was 5.3 h, median cardiac intensive care unit length of stay was 2.0 days, and median postoperative hospital stay was 7.0 days (Table 1).

**Table 1:** Demographic, preoperative, intraoperative, and postoperative clinical characteristics of 118 included children.

Variable	All children(*N*=118)
Age (months)	19.0 (7.0–42.5)
Sex	
Male	60 (50.8)
Female	58 (49.2)
Weight (kg)	10.0 (7.2–16.0)
Height (cm)	80.5 (69.8–103.3)
BMI (kg/m²)	15.2 (14.2–16.3)
Preoperative variables	
Pre–hemoglobin (g/dL)	12.0 ± 1.4
Pre–hematocrit (%)	35.6 (33.7–38.0)
Pre–LVEF (%)	68.0 (65.0–71.0）
Pre–Cyanotic lesions (yes)	12 (10.2)
Diagnosis	
VSD	73 (61.9)
ASD	21 (17.8)
ASD+VSD	7 (5.9)
TOF	3 (2.5)
TAPVC	3 (2.5)
PAPVC	4 (3.4)
DORV	5 (4.2)
TGA	1 (0.8)
CAVSD	1 (0.8)
RACHS–2	1.0 (1.0–1.0)
Intraoperative variables	
CPB duration (min)	84.5 (67.8–101.3)
Surgery duration (min)	240.0 (214.3–272.8)
Aortic cross clamp duration (min)	58.0 (38.0–75.3)
MAP during CPB (mmHg)	51.5 ± 9.2
Temperature during CPB (℃)	32.5 (30.3–33.6)
Urine output during CPB (mL)	30.0 (20.0–60.0)
Hemoglobin during CPB (g/dL)	8.5 (7.8–9.2)
Hematocrit during CPB (%)	25.0 (23.0–27.1)
Postoperative variables	
Extubation time (hour)	5.3 (3.5–45.6)
CICU stay (day)	2.0 (1.0–4.0)
Postoperative hospitalization stay (day)	7.0 (6.0–9.0)
Hospitalization costs (CNY,thousand)	68.8 (56.6–88.4)

BMI, body mass index; LVEF, left ventricular ejection fraction; VSD, ventricular septal defect repair; ASD, atrial septal defect repair; TOF, tetralogy of Fallot repair; TAPVC, total anomalous pulmonary venous connection repair; PAPVC, partial anomalous pulmonary venous connection; DORV, double outlet right ventricle repair; TGA, transposition of the great arteries; CAVSD, complete atrioventricular septal defect; RACHS-2, risk stratification for congenital heart surgery for ICD-10 administrative data; CPB, cardiopulmonary bypass; MAP, mean arterial pressure; CICU, cardiac intensive care unit.

### Serum S100B levels

Serum S100B concentrations followed a consistent perioperative trajectory across the three sampling time point (Figure 2). Levels were lowest at baseline (T0), peaked immediately at the end of surgery (T1), and declined slightly by the first postoperative day (T2). Overall, S100B levels differed significantly across all three time points (*P* < 0.001), with both T1 and T2 values significantly elevated relative to baseline, and T2 values significantly lower than T1 (all *P* < 0.001).

**Figure 2:**
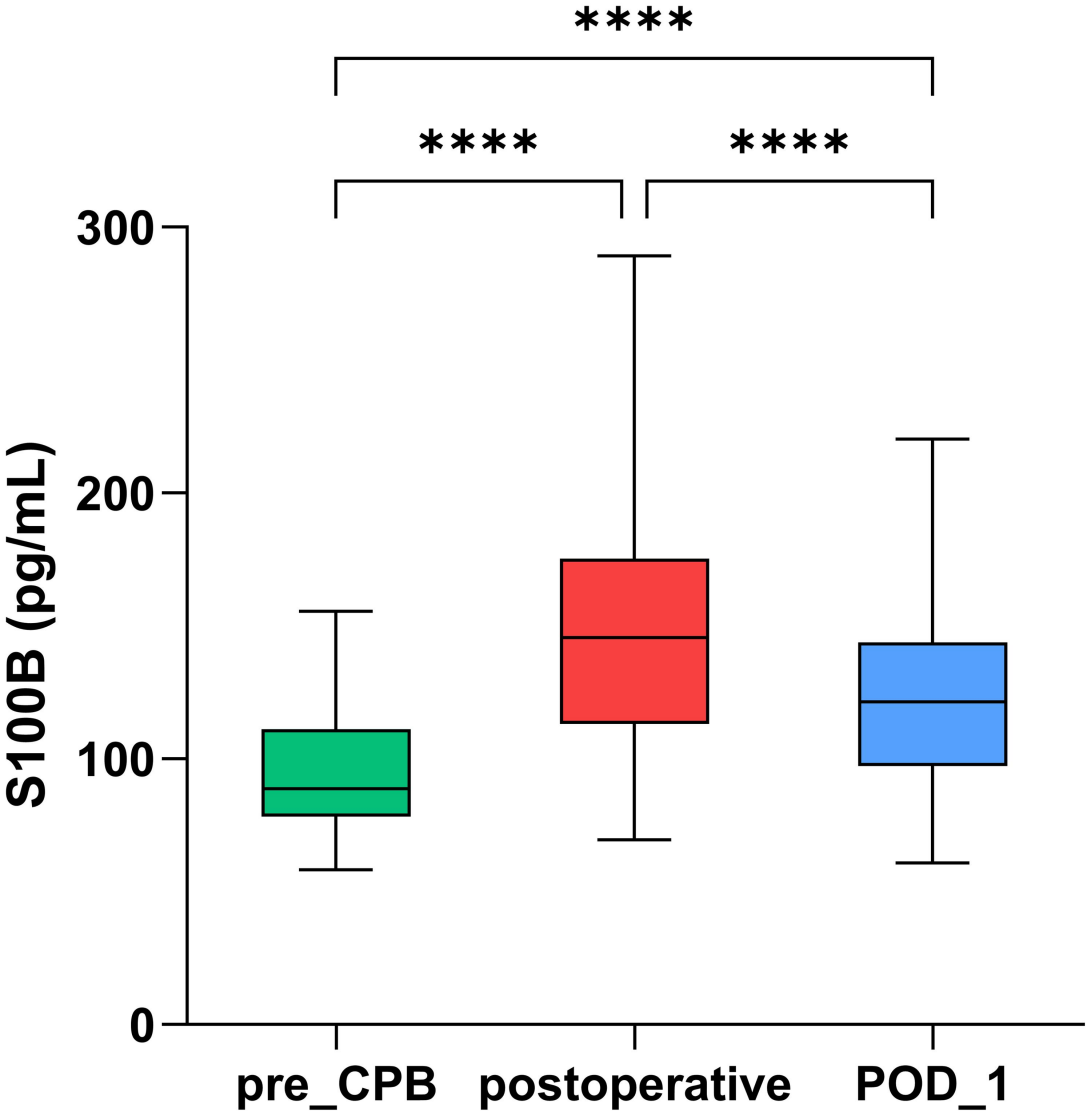
The serum S100B levels at three time points.

### Characteristics of C-rSO_2_

C-rSO₂ parameters exhibited clear phase-dependent variation across the perioperative period (Table 2). Overall, C-rSO₂ values exhibited a clear phase-dependent pattern: they were highest during the pre-CPB period, decreased to their nadir during CPB, and partially recovered in the post-CPB phase. Cerebral desaturation burden, quantified as AUC, was overwhelmingly concentrated during the CPB period across all four relative desaturation thresholds (≥5%, ≥10%, ≥15%, and ≥20% below baseline). As expected, absolute AUC values decreased progressively as the desaturation threshold became more stringent. Per institutional clinical routine, arterial blood gas analyses were systematically performed at scheduled time points during CPB. PaO₂, PaCO₂, and SaO₂ remained stable throughout bypass, with SaO₂ consistently within the prespecified normoxic target range of 95%–98% (Table 2). Spearman correlation analysis revealed a moderate positive correlation between the AUC of C-rSO₂ decrease ≥5% during CPB and total CPB duration (r = 0.58, *P* < 0.001), confirming that longer bypass duration was associated with greater cumulative desaturation burden, consistent with clinical expectations.

**Table 2:** Characteristics of C–rSO_2_ across all anesthetic phases and arterial blood gas values during CPB.

Variable	Overall	Pre–CPB	CPB	Post–CPB
The median value of C–rSO_2_ (%)	78.2 (73.4–81.8)	83.3 (79.4–86.6)	71.7 (66.9–75.4)	80.0 (75.1–84.6)
Desaturation				
AUC for C–rSO_2_ decrease ≥5% (%·min)	193.0 (94.8–305.9)	3.2 (0.9–7.7)	157.9 (80.6–261.6)	24.9 (5.3–50.4)
AUC for C–rSO_2_ decrease ≥10% (%·min)	105.4 (42.8–199.8)	0.1 (0–1.8)	81.8 (29.8–177.0)	8.6 (0.1–28.2)
AUC for C–rSO_2_ decrease ≥15% (%·min)	43.7 (11.1–102.3)	0 (0–0.1)	33.6 (7.3–95.6)	1.7 (0–11.4)
AUC for C–rSO_2_ decrease ≥20% (%·min)	11.3 (0.6–43.9)	0 (0–0)	6.5 (0.1–39.9)	0 (0–2.5)
Arterial blood gas				
Early CPB (5–10 min after initiation)				
PaO₂ (mmHg)	–	–	198 ± 42	–
PaCO₂ (mmHg)	–	–	39 ± 4	–
SaO₂ (%)	–	–	98.2 ± 1.1	–
Mid-CPB (midpoint of cross-clamp)				
PaO₂ (mmHg)	–	–	185 ± 38	–
PaCO₂ (mmHg)	–	–	38 ± 5	–
SaO₂ (%)	–	–	97.9 ± 1.3	–

C–rSO_2_, cerebral regional oxygen saturation; AUC, area under the curve; CPB, cardiopulmonary bypass. PaO₂, partial pressure of arterial oxygen; PaCO₂, partial pressure of arterial carbon dioxide; SaO₂, arterial oxygen saturation.

### Multivariable linear regression analysis

Linear regression models were developed to predict S100B levels at the end of surgery and on the first postoperative day. Across all four relative desaturation thresholds, the association between cumulative desaturation AUC and S100B elevation remained directionally consistent and statistically significant after multivariable adjustment. Model fit decreased monotonically as the desaturation threshold became stricter: adjusted R^2^ declined progressively while AIC and BIC increased, with no peak at an intermediate cutoff (Table 3). This monotonic gradient indicates that the desaturation-S100B association is robust across threshold definitions, and that the ≥5% threshold is the most sensitive metric for capturing cumulative hypoxic burden, rather than representing a discrete pathophysiologic injury cutoff. Accordingly, the threshold comparison is interpreted as a sensitivity analysis rather than a search for a single optimal threshold.

**Table 3:** Comparison of performance metrics for multivariable linear models predicting postoperative S100B levels using cumulative desaturation burden at four relative thresholds.

Models	Adjusted R^2^	AIC	BIC
Models (S100B_postoperative)			
Model_1 (total AUC of C–rSO_2_ decrease ≥5%)	0.75	738.99	763.92
Model_2 (AUC of C–rSO_2_ decrease ≥5% during CPB)	0.80	715.34	740.27
Model_3 (total AUC of C–rSO_2_ decrease ≥10%)	0.71	758.6	783.53
Model_4 (AUC of C–rSO_2_ decrease ≥10% during CPB)	0.76	737.45	762.38
Model_5 (total AUC of C–rSO_2_ decrease ≥15%)	0.64	784.17	809.1
Model_6 (AUC of C–rSO_2_ decrease ≥15% during CPB)	0.68	769.89	794.82
Model_7 (total AUC of C–rSO_2_ decrease ≥20%)	0.57	805.18	830.11
Model_8 (AUC of C–rSO_2_ decrease ≥20% during CPB)	0.60	797.04	821.97
Models (S100B_postoperative day 1)			
Model_1 (total AUC of C–rSO_2_ decrease ≥5%)	0.73	688.62	713.56
Model_2 (AUC of C–rSO_2_ decrease ≥5% during CPB)	0.79	661.88	686.82
Model_3 (total AUC of C–rSO_2_ decrease ≥10%)	0.70	705.01	729.95
Model_4 (AUC of C–rSO_2_ decrease ≥10% during CPB)	0.75	683.59	708.53
Model_5 (total AUC of C–rSO_2_ decrease ≥15%)	0.64	723.17	748.11
Model_6 (AUC of C–rSO_2_ decrease ≥15% during CPB)	0.69	707.77	732.71
Model_7 (total AUC of C–rSO_2_ decrease ≥20%)	0.59	739.15	764.09
Model_8 (AUC of C–rSO_2_ decrease ≥20% during CPB)	0.62	730.05	754.98

C–rSO_2_, cerebral regional oxygen saturation; AUC, area under the curve; CPB, cardiopulmonary bypass.

Three nested sequential models were constructed to quantify the incremental explanatory value of cumulative desaturation burden beyond established predictors (Table 4). Baseline S100B alone explained 31.1% of the variance in T1 S100B. Addition of CPB duration increased adjusted R^2^ to 0.38 (*Δ*R^2^ = 6.9%). Further inclusion of the AUC of C-rSO₂ decrease ≥5% during CPB raised adjusted R^2^ to 0.79, corresponding to an additional 40.5% of variance explained beyond baseline S100B and CPB duration. A similar pattern was observed for T2 S100B, with desaturation AUC contributing an additional 29.6% of explained variance.

**Table 4:** Incremental variance in postoperative S100B explained by cumulative cerebral desaturation burden in nested sequential linear models.

Model specification	Adjusted R² (T1 S100B)	*Δ* adjusted R² (T1)	Adjusted R² (T2 S100B)	*Δ* adjusted R² (T2)
Model 1: Baseline S100B only	0.31	—	0.43	—
Model 2: Model 1 + CPB duration	0.38	0.07	0.49	0.06
Model 3: Model 2 + AUC ≥5% during CPB	0.79	0.41	0.79	0.30

Abbreviation: CPB, cardiopulmonary bypass; AUC, area under the curve.

Univariate and multivariate linear regression analyses were conducted for the two S100B outcome measures, focusing on the AUC of C-rSO₂ decrease ≥5% during CPB as the key predictor. Multivariable linear regression analysis confirmed that the cumulative burden of cerebral oxygen desaturation during CPB emerged as a consistent and independent predictor of elevated S100B levels at both postoperative time points (Table 5). Specifically, the AUC of C–rSO₂ decrease ≥5% during CPB was significantly associated with higher S100B concentrations immediately after surgery (T1: B = 0.22, 95% CI 0.19–0.25, *P* < 0.001) and on the first postoperative day (T2: B = 0.15, 95% CI 0.12–0.18, *P* < 0.001), after adjusting for all other covariates. Each 1%·min increase in the AUC of C–rSO₂ decrease ≥5% was associated with a 0.22 pg/mL increase in S100B immediately after surgery and a 0.15 pg/mL increase on postoperative day 1. To contextualize this effect size within our cohort, the observed IQR for this AUC was 181%·min (80.6–261.6%·min). This translates to an estimated S100B level that is 39.6 pg/mL higher at immediate post-operation and 27.2 pg/mL higher on postoperative day 1 for children at the 75th percentile of desaturation burden compared to those at the 25th percentile. This dose-response relationship underscores that the magnitude of cumulative intraoperative cerebral oxygen desaturation is quantitatively associated with the degree of early postoperative neurological biochemical injury. Full statistical results for all covariates are provided as Supplementary Material. Diagnostic analyses confirmed that the primary multivariable models satisfied linear regression assumptions. Residuals were approximately normally distributed (Shapiro–Wilk *P* = 0.08 for T1, *P* = 0.12 for T2). Residual-versus-fitted plots were generated for all primary models (Supplementary Figure). Visual inspection confirmed that residual variance was fairly constant across the range of fitted values, with no systematic fanning pattern.

**Table 5:** Univariate and multivariate linear regression analyses of factors associated with S100B levels after surgery and on the first postoperative day.

Variable	Models (S100B_postoperative, T1)						Models (S100B_postoperative day 1, T2)					
	Univariable model			Multivariable model			Univariable model			Multivariable model		
	B value	95%CI	*P* value	B value	95%CI	*P* value	B value	95%CI	*P* value	B value	95%CI	*P* value
S100B_preCPB（pg/mL）	1.15	0.84–1.46	＜0.001	1.07	0.75–1.38	＜0.001	1.05	0.83‒ 1.27	＜0.001	1.07	0.82–1.32	＜0.001
Age (months)	−0.85	˗1.12– ˗0.59	＜0.001	−0.05	˗0.29–0.18	0.650	−0.71	˗0.91– ˗0.51	＜0.001	0.06	˗0.13–0.24	0.557
BMI (kg/m²)	−5.74	˗9.52– ˗1.96	0.003	−2.64	˗4.43– ˗0.84	0.004	˗3.28	˗6.28‒ ˗0.28	0.032	−1.02	˗2.46–0.41	0.159
Preoperative variables												
Pre–hemoglobin (g/dL)	−6.29	˗11.90– ˗0.69	0.028	1.86	˗0.92–4.63	0.188	−6.48	˗10.78– ˗2.18	0.003	0.42	˗1.79–2.64	0.706
RACHS–2	14.80	3.71–25.89	0.009	−0.58	˗7.17–6.02	0.863	14.21	5.70–22.73	0.001	0.90	˗4.36–6.16	0.735
Pre–Cyanotic lesions (vs no)	28.55	2.17– 54.93	0.034	−9.70	˗25.04–5.65	0.213	25.91	5.50–46.34	0.013	−12.14	˗24.37–0.09	0.052
Intraoperative variables												
CPB duration (min)	0.51	0.30–0.72	＜0.001	0.08	˗0.06–0.21	0.252	0.41	0.24–0.57	＜0.001	0.06	˗0.04–0.17	0.230
AUC of C–rSO_2_ decrease ≥5% during CPB (%·min)	0.25	0.21–0.29	＜0.001	0.22	0.19–0.25	＜0.001	0.17	0.14–0.21	＜0.001	0.15	0.12–0.18	＜0.001

CI, confidence interval; BMI, body mass index; RACHS-2, risk stratification for congenital heart surgery for ICD-10 administrative data; CPB, cardiopulmonary bypass; C–rSO_2_, cerebral regional oxygen saturation; AUC, area under the curve.

### Sensitivity analysis

A series of prespecified sensitivity analyses were performed from multiple dimensions to evaluate the robustness of the primary association between the AUC of C–rSO₂ decrease ≥5% during CPB and T1 S100B elevation, all yielding consistent results.

First, to address potential confounding by intraoperative clinical parameters, we sequentially added prespecified confounders (aortic cross-clamp time, mean CPB hemoglobin, mean CPB MAP, and mean CPB temperature) to the base model. The effect size of desaturation AUC remained highly stable across all stepwise models, with *β* coefficients ranging from 0.212 to 0.219 and all retaining statistical significance (all *P* < 0.001). The fully adjusted model showed a less than 3% change in effect size relative to the base model (Table 6).

**Table 6:** Stepwise sensitivity analyses for the association between AUC of C–rSO_2_ decrease ≥5% during CPB and postoperative T1 S100B concentration.

Model specification	Adjusted covariates	*β* coefficient	95% CI	*P* value
Model 1: Base model	Baseline S100B	0.219	0.186–0.252	＜0.001
Model 2	Base model + aortic cross-clamp time	0.217	0.184–0.250	＜0.001
Model 3	Model 2 + mean CPB hemoglobin	0.215	0.182–0.248	＜0.001
Model 4	Model 3 + mean CPB MAP	0.214	0.181–0.247	＜0.001
Model 5	Model 4 + mean CPB temperature	0.212	0.177–0.247	＜0.001

CPB, cardiopulmonary bypass; AUC, area under the curve; MAP, mean arterial pressure.

Second, to resolve the mathematical coupling between cumulative AUC and total CPB duration, we constructed a time-normalized desaturation rate metric (AUC divided by CPB duration, unit: %), representing the average relative desaturation intensity per unit time. This time-normalized metric remained independently associated with T1 S100B (*β* = 18.6, *P* < 0.001), confirming that the association is driven by desaturation severity rather than simply longer bypass time.

Third, we tested the impact of baseline definition using a restricted 5-minute steady-state window 10–15 min after intubation. The correlation between the full pre-CPB baseline and the restricted stable baseline was r = 0.94, and the effect size of the AUC of C–rSO₂ decrease ≥5% during CPB on T1 S100B was nearly identical (*β* = 0.21 vs. 0.22), indicating that baseline selection did not materially affect the results.

Additionally, further adjustment for mean CPB PaO₂ did not substantially alter the association (adjusted β = 0.21, 95% CI 0.18–0.24, *P* < 0.001), excluding the possibility that arterial oxygen tension was a major confounding factor.

Finally, we re-specified the model using natural log-transformed T1 S100B as the dependent variable to assess scale dependency. For T1, the coefficient for the AUC of C-rSO₂ decrease ≥5% during CPB was 0.0015 (95% CI 0.0012–0.0017, *P* < 0.001) on the natural log scale, corresponding to an approximate 0.15% increase in S100B per 1%·min increase in AUC. For T2, the corresponding coefficient was 0.0012 (95% CI 0.0010–0.0014, *P* < 0.001). The direction and statistical significance were unchanged across both additive and multiplicative specifications, confirming the robustness of the primary findings.

### Optimal cutoff of cumulative cerebral desaturation burden

ROC curve analysis showed that the cumulative AUC of C-rSO₂ decrease ≥5% during CPB had good predictive value for elevated postoperative peak S100B (defined as above the 75th percentile of the cohort, i.e., 175.43 pg/mL), with an AUROC of 0.87 (95% CI: 0.78–0.97, *P* < 0.001). The optimal cutoff value was 217.1%·min, yielding a sensitivity of 86.2% and a specificity of 84.3% (Youden's index = 0.705) (Figure 3). Bootstrap internal validation with 1,000 resamples yielded an optimism-corrected AUROC of 0.84 (95% bootstrap percentile CI: 0.75 to 0.92). At the resample-derived optimal cutoff, the bootstrap-corrected sensitivity was 82.1% (95% bootstrap CI: 72.8% to 89.7%) and the bootstrap-corrected specificity was 80.4% (95% bootstrap CI: 71.1% to 88.3%). The optimal cutoff was re-derived independently in each bootstrap iteration. The median cutoff across all 1,000 resamples was 210.8%·min, with a 95% percentile confidence interval of 183.5%·min to 248.2%·min. The relatively narrow range of variation indicates that the derived cutoff is reasonably stable across resamples. The relatively small magnitude of optimism correction (approximately 3% for AUROC) suggests that the original performance estimates were not severely inflated.

**Figure 3:**
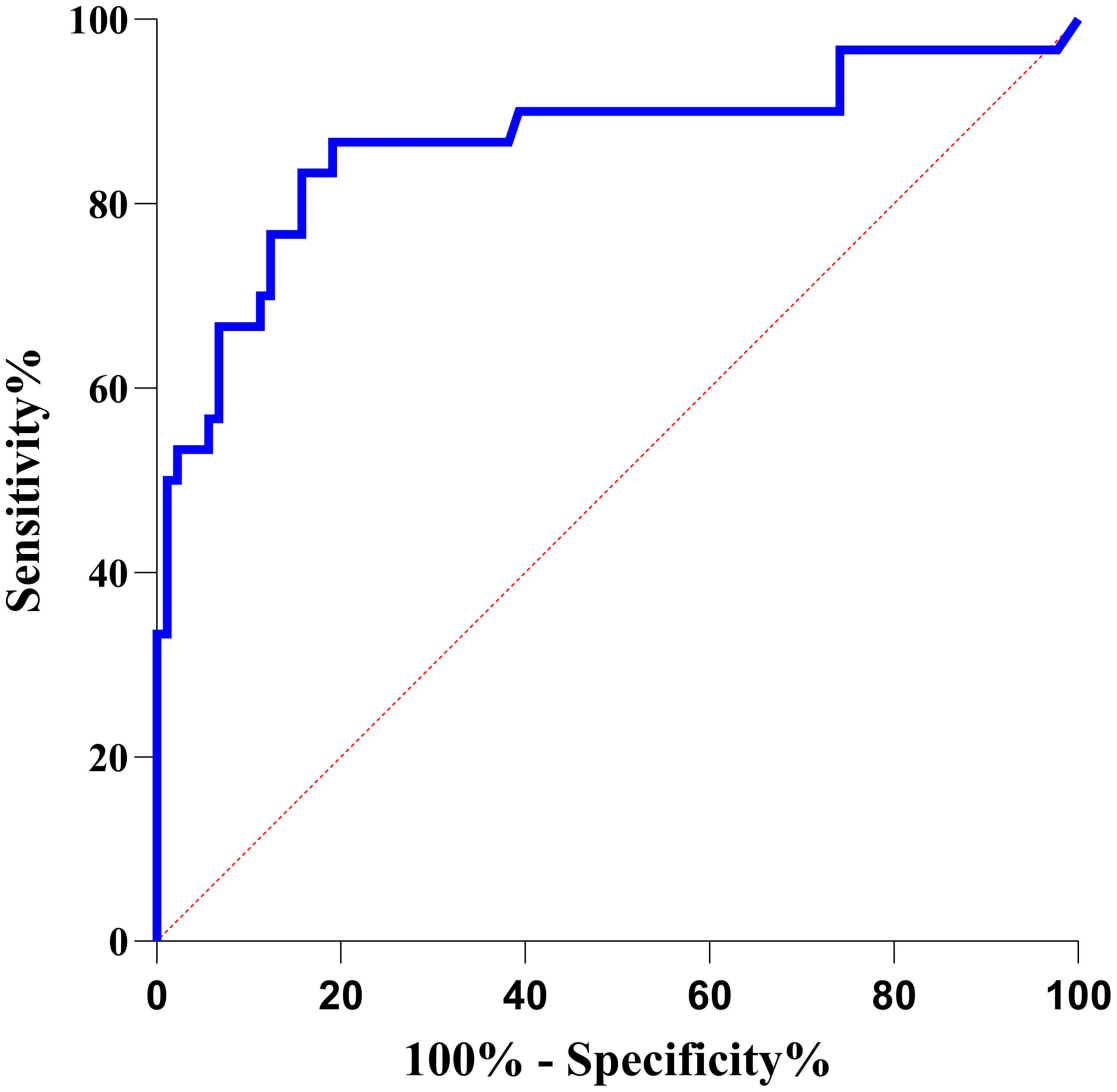
Receiver operating characteristic curve of cumulative cerebral desaturation burden for predicting elevated serum S100B at the end of surgery.

### Exploratory analysis with clinical outcomes

Of the 118 enrolled children, 8 (6.8%) experienced at least one clinical seizure during the postoperative hospital stay. No seizure-related mortality occurred (Table 7). Children who developed seizure had significantly higher serum S100B levels at T1and T2 compared to those without seizure (T1, 213.5 [190.4–233.1] vs. 142.9 [109.2–169.3] pg/mL, *P* < 0.001; T2, 169.4 [161.8–185.8] vs. 118.3 [94.9–137.1] pg/mL, *P* = 0.001). Similarly, the AUC of C-rSO₂ decrease ≥5% during CPB was significantly larger in the seizure group [416.1 [411.8–512.5] vs. 151.4 [78.8–233.4] %·min, *P* < 0.001].

**Table 7:** Comparison of cumulative cerebral desaturation burden and perioperative S100B levels between children with and without postoperative clinical seizures after congenital heart surgery.

Variable	Seizure (*n*=8)	No seizure (*n*=110)	*p*-value
AUC of C-rSO₂decrease ≥5% during CPB (%·min)	416.1 (411.8–512.5)	151.4 (78.8–233.4)	＜0.001
S100B at baseline (pg/mL)	102.6 (86.5–122.0)	88.4 (78.2–109.7)	0.179
S100B at end of surgery (pg/mL)	213.5 (190.4–233.1)	142.9 (109.2–169.3)	＜0.001
S100B at POD 1 (pg/mL)	169.4 (161.8–185.8)	118.3 (94.9–137.1)	0.001

AUC, area under the curve; C–rSO_2_, cerebral regional oxygen saturation; CPB, cardiopulmonary bypass.

The relationship between postoperative serum S100B levels and relevant clinical outcomes was assessed using Spearman's rank correlation. Serum S100B levels at T1 showed significant positive correlations with the duration of postoperative mechanical ventilation (r = 0.44, *P* < 0.001), total hospital length of stay (r = 0.31, *P* < 0.001), and total hospitalization costs (r = 0.40, *P* < 0.001).

## Discussion

This prospective observational cohort study demonstrates that the cumulative duration and severity of intraoperative cerebral desaturation are independently associated with elevated postoperative S100B, a sensitive biochemical marker of early cerebral injury, even after adjusting for established surgical and clinical risk factors. Establishing a linkage between intraoperative NIRS parameters and neurologic outcomes in children is challenging owing to developmental constraints in neurobehavioral testing. This limitation underscores the clinical significance of non-invasive, continuous NIRS monitoring as an objective tool for assessing cerebral oxygenation status, particularly in its capacity to predict neurotrauma-associated serum biomarkers such as S100B.

In this cohort of 118 children, serum S100B levels followed a characteristic perioperative pattern. Median concentrations were lowest at the preoperative baseline after induction of anesthesia, peaked immediately post-surgery, and subsequently showed a partial decline by the first postoperative day in the cardiac intensive care unit. This pattern indicates a systemic release of S100B in response to surgical and perfusion-related stress, with levels remaining elevated during early recovery. Our findings are consistent with previous reports of a perioperative rise in S100B, starting with the CPB initiation, peaking after its cessation, and gradually decreasing thereafter (18, 19). Postoperative clinical seizure (defined as any acute, self-limited, abnormal involuntary movement or alteration of consciousness documented by the attending physician as “seizure,” “convulsion,” or “suspected seizure”) occurred in 8 children (6.8%), with no seizure-related mortality. All children were successfully discharged without major complications. Exploratory analysis further revealed that elevated postoperative S100B levels correlated significantly with prolonged mechanical ventilation, extended hospital stays, and increased total hospitalization costs. These preliminary findings suggest that the magnitude of perioperative neurological stress, as reflected by S100B elevation, may be associated with perioperative complications, thereby contributing to increased clinical resource utilization. This potential link warrants validation in future studies designed with clinical outcomes as primary endpoints. Our results are consistent with and extend the findings of prior similar investigations (20–22).

C-rSO₂ exhibited phase-dependent variations throughout the anesthetic course. While the overall median value was 78.2%, a clear temporal pattern was observed: values were highest before CPB (83.3%), declined to their lowest level during CPB (71.7%), and showed partial recovery thereafter (80.0%). A notable finding was the substantial burden of cerebral desaturation, quantified by the AUC, which occurred predominantly during CPB. This pattern was consistent across all desaturation thresholds (≥5%, ≥10%, ≥15%, and ≥20%), with the corresponding AUC gradually diminishing as the threshold increased. Collectively, these findings identify CPB as the period of greatest vulnerability for cerebral oxygenation in this pediatric cardiac surgical cohort.

Among regression models evaluating the association between selected cerebral oxygenation variables (with only one variable included per model) and postoperative S100B levels, the AUC for C–rSO₂ desaturation ≥ 5% during CPB was the strongest predictor (Table 3). This model demonstrated the highest adjusted R^2^ and the best model fit, as evidenced by the lowest AIC and BIC values. This dose-response gradient carries important pathophysiological implications: it suggests that cumulative hypoxic exposure, rather than isolated severe desaturation events, drives glial activation and S100B release, supporting the clinical value of monitoring total desaturation burden rather than only extreme single-point values. It supports the concept that even sustained mild desaturation contributes to cumulative cerebral stress, rather than injury being driven solely by rare severe desaturation events. This quantitatively links the intraoperative cerebral desaturation burden to the magnitude of potential early neurobiochemical injury. These findings carry important pathophysiological and clinical implications. The observed association aligns with the known role of S100B as a biomarker released upon neuronal and astrocytic injury (23–26). The observed association between cumulative C–rSO₂ desaturation during CPB and S100B elevation is biologically plausible: cerebral ischemic-hypoxic stress may induce blood-brain barrier dysfunction and astrocyte activation, which could contribute to increased circulating S100B concentrations. This study aims to reveal a dynamic dose-response relationship between this quantifiable “hypoxic burden” and a specific biochemical marker of brain injury in pediatric cardiac surgery using high-resolution cerebral oximetry. This supports the concept that advanced analysis of C–rSO₂ monitoring data, such as calculating the AUC for a defined desaturation threshold, extends beyond real-time physiological reflection and can serve as an early, quantitative proxy for subclinical neurological injury. Consequently, such metrics may help identify a potential window for intervention prior to the manifestation of irreversible neurological deficits.

Consistent with our primary hypothesis, cumulative cerebral desaturation burden during CPB was independently associated with elevated postoperative S100B concentration in this pediatric cohort. This association remained statistically significant and materially unchanged after further adjustment for prespecified clinically relevant confounders, including aortic cross-clamp time, mean CPB hemoglobin, mean CPB arterial pressure, and mean CPB temperature. The stability of the effect size across models indicates that the observed relationship is not driven by differences in surgical complexity, hemodilution, cerebral perfusion pressure, or body temperature, supporting the robustness of our primary finding. Besides, the association remained significant when using a time-normalized desaturation rate metric, which eliminates the mathematical influence of CPB duration.

Exploratory ROC curve analysis further corroborated the robust predictive performance of this cumulative cerebral desaturation metric, yielding an AUROC of 0.87 (95% CI: 0.78–0.97, *P* < 0.001) for identifying elevated postoperative S100B peak levels. The exploratory cutoff of 217.1%·min, derived internally from the study cohort, achieved a sensitivity of 86.2% and a specificity of 84.3% (Youden's index = 0.705). However, this value should be interpreted as an exploratory and preliminary estimate rather than a definitive clinical warning threshold. It is intended primarily for hypothesis generation and sample size planning in future prospective validation studies, and requires confirmation in independent external cohorts before any clinical application.

Research on cerebral oximetry has employed standardized absolute thresholds to define clinically relevant desaturation. For instance, studies in infants have used fixed values such as 40% or 50% to categorize cerebral oxygen desaturation (19). However, applying such fixed values fails to account for significant physiological variability between children, including differences between cyanotic and acyanotic circulations, the hemodynamic effects of intracardiac shunts, and age-dependent influences on baseline cerebral oxygenation in the developing brain. In adults, a reduction of ≥20% from baseline C-rSO₂ is considered detrimental to brain function (27). Corresponding evidence suggests that in children, whose brains are still developing, a decrease below 20% of baseline may also be associated with adverse neurological outcomes (28, 29). Therefore, we defined desaturation based on relative percentage reductions from each child's individual baseline (from 5% to 20%, in 5% intervals). This threshold, integrated with the duration of desaturation, was used to calculate the AUC as our primary exposure metric. This approach provides a more comprehensive characterization of cerebral hypoxic burden by accounting for both the depth and persistence of desaturation events. It is particularly relevant for children with CHD, a population characterized by heterogeneous physiological baselines. Our analysis confirms that the metrics derived from this relative method exhibit superior robustness in predicting biochemical injury, thereby providing a novel methodological rationale for optimizing early-warning thresholds in cerebral oximetry. In this study, the AUC for C–rSO₂ desaturation ≥ 5% during CPB showed the best predictive performance, which may be attributed to two reasons. First, S100B is a sensitive early biomarker of astrocyte injury, and mild but sustained cerebral hypoxia can induce astrocyte activation and blood-brain barrier damage, leading to S100B release. Second, severe desaturation (≥15% or ≥20% below baseline) had a low incidence and small AUC values in this cohort, resulting in large data variability and limited predictive ability. Our findings suggest that even mild cerebral desaturation should be valued during pediatric CPB, and cumulative hypoxic burden is a more sensitive indicator than extreme single-point values.

Postoperative clinical seizure occurred in 6.8% of our cohort, consistent with published rates. Children with seizure had significantly higher S100B at T1 and T2 and a substantially larger AUC of cerebral desaturation. In exploratory analyses, children who developed postoperative seizures exhibited substantially greater desaturation burden and higher S100B levels compared with those without seizures. These findings are consistent with a potential dose-response gradient, but given the low event rate and absence of multivariable adjustment, this analysis remains strictly hypothesis-generating. Larger prospective studies are needed to confirm whether cumulative desaturation burden and S100B elevation carry independent prognostic value for clinical seizure risk.

From a methodological perspective, the baseline C-rSO₂ measurements in this study were uniformly acquired under general anesthesia with 100% FiO₂ across the full pre-CPB period from endotracheal intubation to bypass initiation. This standardized ventilation setting, paired with the relatively long baseline window, ensures consistent measurement conditions across all participants and strengthens internal comparability of relative desaturation metrics. A sensitivity analysis using a narrower, hemodynamically stable 5-minute window 10–15 min after intubation confirmed that the inclusion of immediate post-intubation data did not materially alter our primary findings, with a correlation of r = 0.94 between the two baseline definitions and nearly identical effect sizes for the main association. That said, the baseline value represents an anesthetized, hyperoxic reference rather than a physiological awake baseline in room air, which should be taken into account when interpreting the relative desaturation thresholds derived from this cohort.

The primary analysis was performed on the absolute (additive) scale of S100B concentration, which aligns with conventional clinical interpretation of biomarker levels. However, as with most circulating biomarkers, S100B exhibits right-skewed distributions and increasing variability at higher concentrations, raising the question of whether proportional effects might be more generalizable. Sensitivity analysis using natural log-transformed S100B confirmed that the association between cumulative desaturation AUC and postoperative S100B elevation was robust to scale choice. Direction and statistical significance were preserved on both additive and multiplicative scales. On the log scale, each 1%·min increase in AUC of C-rSO₂ decrease ≥5% corresponded to an approximate 0.15% relative rise in T1 S100B. This proportional estimate is complementary to the absolute effect: the additive value informs threshold-based clinical decision-making, while the proportional estimate better supports cross-population generalizability where baseline S100B levels may differ systematically. Diagnostic assessments further validated the primary model specification. The residual-versus-fitted plots demonstrated no fanning pattern, indicating that inclusion of baseline S100B as a covariate adequately addressed the skewed nature of raw biomarker values.

Threshold sensitivity and biological implications. The monotonic decrease in model performance across stricter desaturation thresholds—with no peak at an intermediate cutoff—provides insight into the nature of desaturation-related S100B release. This pattern does not support the existence of a discrete depth threshold below which astrocytic injury abruptly occurs. Instead, it suggests that cumulative hypoxic exposure, even at mild degrees of desaturation, drives S100B elevation. The superior sensitivity of the ≥5% cutoff indicates that the total duration of even subtle cerebral desaturation contributes meaningfully to biochemical brain injury, rather than injury being driven solely by rare deep desaturation events. This aligns with the understanding that sustained mild hypoxia can induce astrocyte activation and blood-brain barrier permeability, contributing to S100B release into the circulation.

This study has several limitations. First, as an observational study, residual confounding may persist despite multivariate adjustment. Second, while S100B is a sensitive biomarker of neurological injury, it lacks specificity and can be elevated in response to systemic inflammation. Third, the monotonic performance gradient across thresholds may partly reflect the relatively low prevalence of severe desaturation events in our cohort; the relative performance of deeper thresholds may differ in populations with more profound and prolonged hypoxic episodes. Fourth, our institution-specific standardized oxygenation protocol (FiO₂ 1.0 during pre-/post-CPB ventilation; normoxic CPB targeting SaO₂ 95%–98%). As oxygenation settings directly affect cerebral oximetry readings and desaturation burden calculations, our findings should be generalized cautiously to centers with different intraoperative oxygenation strategies. Finally, our findings establish a correlation between cerebral oxygenation parameters and S100B as an intermediate biochemical endpoint. The observed association in this study reflects a link between intraoperative desaturation burden and postoperative biochemical cerebral stress, and cannot be equated to a proven causal effect on clinically manifest brain injury. All analyses of clinical neurological outcomes (including postoperative seizures) and secondary resource utilization endpoints were exploratory and hypothesis-generating by design. Formal validation in larger cohorts with standardized neuroimaging, electroencephalography, and long-term neurodevelopmental assessments is required to establish the clinical prognostic value of cumulative desaturation burden.

## Conclusion

In pediatric cardiac surgery with cardiopulmonary bypass, the cumulative burden of C-rSO₂ decrease ≥5% from individual baseline during CPB is robustly and independently associated with early postoperative S100B elevation, with a significant dose-response relationship.

## Data Availability

The original contributions presented in the study are included in the article/Supplementary Material, further inquiries can be directed to the corresponding authors.
