# Supplementary material for: Cumulative cerebral desaturation burden during cardiopulmonary bypass predicts postoperative S100B elevation in pediatric cardiac surgery: a prospective cohort study
Source: Front Pediatr. 2026 Sep 14;14:1922477. doi: 10.3389/fped.2026.1922477 (PMC13617039; doi:10.3389/fped.2026.1922477)
Supplement: Supplementary file 1 [file Supplementaryfile1.docx]

| Supplementary table. Comparison of statistical parameters for variable AUC of C–rSO_2_ desaturation in multivariate linear regression models. | | | | | |  |  |
| --- | --- | --- | --- | --- | --- | --- | --- |
|  | Models (S100B_postoperative) | | | Models (S100B_postoperative day 1) | | |  |
| Models | B value | 95%CI | *P* value | B value | 95%CI | *P* value |  |
| Model_1 (total AUC of C–rSO_2_ decrease ≥5%) | 0.15 | 0.13–0.18 | ＜0.001 | 0.10 | 0.08‒0.12 | ＜0.001 |  |
| Model_2 (AUC of C–rSO_2_ decrease ≥5% during CPB) | 0.22 | 0.19–0.25 | ＜0.001 | 0.15 | 0.12–0.18 | ＜0.001 |  |
| Model_3 (total AUC of C–rSO_2_ decrease ≥10%) | 0.17 | 0.14–0.21 | ＜0.001 | 0.11 | 0.08‒0.14 | ＜0.001 |  |
| Model_4 (AUC of C–rSO_2_ decrease ≥10% during CPB) | 0.25 | 0.21–0.29 | ＜0.001 | 0.17 | 0.13‒0.20 | ＜0.001 |  |
| Model_5 (total AUC of C–rSO_2_ decrease ≥15%) | 0.18 | 0.13–0.24 | ＜0.001 | 0.12 | 0.08–0.16 | ＜0.001 |  |
| Model_6 (AUC of C–rSO_2_ decrease ≥15% during CPB) | 0.27 | 0.20–0.33 | ＜0.001 | 0.18 | 0.13–0.23 | ＜0.001 |  |
| Model_7 (total AUC of C–rSO_2_ decrease ≥20%) | 0.18 | 0.11–0.25 | ＜0.001 | 0.11 | 0.05–0.16 | ＜0.001 |  |
| Model_8 (AUC of C–rSO_2_ decrease ≥20% during CPB) | 0.28 | 0.18–0.37 | ＜0.001 | 0.18 | 0.11‒0.25 | ＜0.001 |  |
| Abbreviation: CI, confidence interval; CPB, cardiopulmonary bypass; C–rSO_2_, cerebral regional oxygen saturation; AUC, area under the curve. | | | | | | |  |
|  |  |  |  |  |  |  |  |

**Supplementary Figure.** Residuals versus fitted values plot for the primary multivariable model. No systematic pattern or heteroscedasticity was observed.
